# The sex change of the caridean shrimp *Hippolyte inermis* Leach: temporal development of the gonopore morphology

**DOI:** 10.1007/s00435-018-0405-z

**Published:** 2018-04-20

**Authors:** Mirko Mutalipassi, Chingoileima Maibam, Valerio Zupo

**Affiliations:** Functional and Evolutionary Ecology Laboratory, Stazione Zoologica Anton Dohrn, Punta San Pietro, 80077 Ischia, Italy

**Keywords:** Sex reversal, *Appendix interna*, *Appendix masculina*, Gonopore, Protandric hermaphroditism

## Abstract

Sex reversal is a process observed in several marine organisms, including some lineages of caridean shrimps. We investigated the gonopore shape and size, to study the sex reversal using *Hippolyte inermis* as a model. A method was developed which can be applied to identify the sex in juveniles of *H. inermis*, especially, useful when the standard method of sex assessment is not applicable. The position and the shape of gonopores was recorded under a light macroscope. The sex of mature individuals was then determined by observing the presence/absence of the *appendix masculina*. In addition, analysis of ontogenetic changes of gonopores were performed to compare their morphology with other species of shrimps whose gonopore morphology was previously known. Female gonopores are located at the far proximo-medial end of the third pair of pereiopod coxae and distally they bear cup-shaped structures, whilst male gonopores are located at the far proximo-medial end of the fifth pair of pereiopod coxae and they have a simpler structure. The shape and structure of gonopores in *H. inermis* resembled that of other caridean decapods. Intersex individuals were never observed, although this species was demonstrated to be protandric. This observation confirmed previous assumptions indicating that the process of sex reversal is very fast in *H. inermis* and that it takes place within a single moult. The identification of sex based on the position and shape of gonopores is feasible in this species, and it provides helpful insights for studying sex reversal in small decapods.

## Introduction

Caridean shrimps exhibit a range of sexual systems and several investigations clarified details of their maturation and reproduction (Gherardi and Calloni 1993; Bauer 2000; Bergström 2000; Lin and Zhang 2001; Chockley and St. Mary 2003; Zhang and Lin 2005; Baeza 2006; Zupo et al. 2008). In particular, according to the tissue persistence of the androgenic gland, various sexual strategies were observed in a gradient of cases, from total stability of androgenic gland (persistence of testes in gonochoristic species) to fast sex reversal as observed in *H. inermis* (Table 1; Zupo and Maibam 2010).

**Table 1:** Various sexual strategies as exhibited by caridean shrimps: (a) typical gonochoristic species, bearing a persistent AG, (b) *Lysmata*-type contemporaneous hermaphrodites, (c) *Processa*-type protandric species, (d) *Hippolyte inermis* and it’s very low-persistence AG. Scheme according to Zupo and Maibam (2010)

	Typology	Persistence of AG tissues	Intermediate stage	Final stage
Case a	*Penaeus* type	High	Testis	Testis
Case b	*Lysmata* type	Medium	Ovotestis	Ovotestis
Case c	*Hippolyte niezabitowskii* type	Low	Ovotestis	Ovary
Case d	*Hippolyte inermis* type	Very low	Ovary	Ovary

*Hippolyte inermis* is a protandric consecutive hermaphrodite shrimp (Reverberi 1950; Veillet et al. 1963; Yaldwyn 1966), distributed from Ireland and Western Channel to Morocco (O’Céidigh 1962; Murray 1980), Sea of Marmara (d’Udekem d’Acoz 1996) and Mediterranean seagrass meadows (Zariquiey Alvarez 1968; Guillén 1990; d’Udekem d’Acoz 1998), in particular, *Posidonia oceanica* (Gambi et al. 1992). Two periods of recruitment have been observed in the field (Zupo 1994). The first reproductive period occurs in spring and yields offspring consisting of both males and females. The second occurs in fall, and offspring are characterised by males that undergo sex reversal after the next spring recruitment period (Veillet et al. 1963). In fact, *H. inermis* is characterised by sex reversal that proceeds through complete regression of the male gonad and development of an ovary from undifferentiated germinal cells (Reverberi 1950), without passing through a transitional stage of “ovotestis” (Cobos et al. 2005) as commonly observed in decapod crustaceans (Bauer and Holt 1998). Another feature making the reproductive biology of this species unique is that, besides the well-known sex reversal process observed in individuals aged approximately 1 year, as first described by Reverberi (1950), an additional mechanism may provide young females. Individuals born in spring and feeding on diatoms of the genus *Cocconeis*, dominating the microphytic community in that period, show early sex reversal and the production of small (6–7 mm) females (Zupo 1994). These females are produced due to the early regression of the androgenic glands in juveniles, subsequent to feeding on diatoms. Herein, and further in the text, we use the term “juvenile” to specify the stages that occur after the last zoel stage, inclusive of all developmental stages to the mature adult as according to Felder et al. (1985) and with abdominal propulsion (Gurney 1942). Therefore, both large (more than 12 mm and named “alpha”) and small (less than 9 mm and named “beta”) females are present in natural populations (Zupo 1994). The first are produced by 1-year-old males, due to a progressive ageing of their androgenic glands; the latter are produced by juveniles of about 1–2 months, when they feed on selected diatoms that trigger an early apoptosis of their androgenic glands and testes (Zupo and Messina 2007).

Often, the sex of decapod crustaceans is assessed by studying the morphology of pleopods (presence/absence of a male appendix named *appendix masculina, A.M*.) using a dissecting microscope, especially when the species of concern is small and the characters used for discrimination of sexes are easy to observe. However, this method is not applicable when said characters are still in immature stages or hardly detectable. In such cases, gonopore shape and location can be used as an alternative for sex assessment (Tóth and Bauer 2007). Size, shape and position of gonopores are not often used to identify the sex of decapod crustaceans because of the difficulty to detect the pores, especially in small individuals. However, it has been used when the standard method of sex assessment based on the presence/absence of the male appendix on the endopodite of the second pair of pleopods (as observed in *H. inermis*), was not applicable.

One such case of difficult observation of gonopores using a dissecting microscope was reported by Dardeau (1984) for species of snapping shrimps, *Synalpheus* spp. Nevertheless, in some species, the sex identification is possible and easy. For example, Mascetti et al. (1997) described the morphological structures of the male and female gonopores in the Antarctic hippolytid shrimp, *Chorismus antarcticus*. In addition, the size range at which some protandric species change from male to female was demonstrated, thanks to this technique. In addition, in a deep-sea sponge-associated shrimp, *Spongicola japonica*, the appearance of gonopores was used as a first indication of sexual dimorphism in both sexes (Saito 2002). In *Synalpheus* spp., the standard technique of sex assessment is not applicable, due to the absence of an *A.M*. (Banner and Banner 1975; Felder 1982; Dardeau 1984). Tóth and Bauer (2007) used the gonopore technique for “helper” individuals, in a *Synalpheus* colony, to distinguish between males, non-reproductive females and juveniles. Tóth and Bauer (2008) also analysed the sex ratio of the colony members and their queens in two species of *Synalpheus*.

In *H. inermis* the standard method of sex assessment, i.e., the detection of the presence of an *A.M*., is applicable only after an individual has undergone sexual maturation, about 1–2 months after the larval settlement (Zupo et al. 2008). The sexual appendices, i.e., *appendix masculina* and *appendix interna* (*A.I*.), are underdeveloped or absent in the juveniles, hence, it is impossible to determine their sex using this method. For this reason, the assessment of sex might be performed, in immature individuals, by detecting the location and shape of gonopores.

*Hippolyte inermis* is a model organism for the investigation of apoptogenic compounds (Zupo and Messina 2007; Zupo et al. 2014) and for studies on the mechanisms of sex reversal in decapods (Zupo and Maibam 2010; Cobos et al. 2011). Therefore, an early assessment of sex in juveniles could be of benefit. As the external sexual characters appear, in this species, in 30–50-days-old individuals and the process can be affected by several factors (e.g., food quality, presence of bacteria, water quality and maternal influences) in cultured individuals, an additional method, applicable for younger juveniles, is useful. As a matter of fact, the assessment of sex based on the shape of gonopores was applied only to selected species of decapod crustaceans (Mascetti et al. 1997; Saito 2002; Tóth and Bauer 2007, 2008) and previous studies indicated clear differences in the ultra-structure and the patterns of maturation of gonopores in each species. Therefore, the description of these secondary sexual features in this peculiar hippolytid shrimp may add information, worth to study the whole hippolytidae taxon. For these reasons, we have taken into account both sexually immature and mature individuals, to investigate the position of gonopores and their morphology, using a complanar macroscope. In addition, when possible (larger individuals), we determined the sex of mature individuals by means of the presence/absence of *A.M*., to confirm the accuracy of our conclusions.

## Methods

### Sample collection

Ovigerous females of *H. inermis* Leach, 1815, were collected in Lacco Ameno d’Ischia (Gulf of Naples, Italy) at depths of 3–15 m. A *P. oceanica* meadow extends continuously from 1 to about 33 m depth in this area (Mazzella and Buia 1989), and previous studies on the population dynamics and the sex reversal of *H. inermis* were accomplished on individuals collected in the same area (Zupo 1994). The collections were performed at noon, by towing a plankton net (400 mm diameter; mesh size 100 µm) horizontally across a *Posidonia* meadow, from a boat at a speed of about 3.7 km h^−1^. Each collection consists of a 3–5 min tow, to avoid clogging the net with seagrass leaves that could hurt the collected individuals. The first sorting was performed visually, and shrimps were stored in a container (300 × 400 mm) filled with 30 mm of seawater (38 psu). In the laboratory, after species identification, each *H. inermis* female was individually transferred in a conical flask filled with 1.5 L of filtered seawater, aerated by means of an air pump connected to a plastic tube. Nauplii of *Artemia* cf. *franciscana* (Coppens^®^ premium cysts, 3 individuals/mL) were added to the seawater (38 psu) and the conical flasks were kept in a thermostatic chamber (18 °C) until the release of *H. inermis* larvae. Culture vessels were checked every morning for the presence of larvae, indicated also by the occurrence of adult female exuviae (Zupo and Messina 2007), since the release of larvae corresponds with exuviation of ovigerous females. When larvae were found, they were sieved through a 60 µm filter, counted and transferred to their rearing vessels.

### Larval rearing

Collected larvae were cultured under laboratory conditions until they reached the juvenile stage, which occurred within 18–22 days under our experimental conditions (Zupo 2000). These involve culturing larvae in 1 L conical flasks, each containing 800 mL of culture medium. The larval phase consists of eight stages and has variable development time depending on culture conditions (Lebour 1931; Le Roux 1963; Zupo and Buttino 2001). The size of the zoea ranges from 1 to 1.6 mm (first zoel stage) to 3.0–4.0 mm (last zoel stage, Zupo and Buttino 2001). Larvae were fed *ad libitum* on nauplii of *Artemia franciscana* (four nauplii of freshly hatched Coppens^®^ premium cysts per mL) along with the rotiferid *Brachionus plicatilis* (4 ind/mL) for the first 7 days. From the 8th day onwards, enriched *A. franciscana* nauplii were used instead of freshly hatched nauplii, at the same density. For enrichment, 24 h old nauplii were kept in enrichment media (AlgaMac-2000, Aquafauna Bio-Marine, Inc) for at least 12 h. After 24 h of enrichment, the nauplii were harvested. Enriched nauplii were added at the same rate during the whole larval growth, whilst the number of *Brachionus* was reduced to 4, 3 and 2 ind/mL on the 9th, 10th and 11th day, respectively. *Brachionus* administration was stopped from the 12th day onwards.

### Rearing of juveniles

Larvae that changed into juveniles were transferred into deep-walled Petri dishes of 500 mL at a density of 1 juvenile/16 mL of filtered and sterilised seawater (25 juveniles in 400 mL of seawater 38 psu) and were cultured until they reached sexual maturation, under the same conditions as mentioned above. A maximum of 5 days was needed to complete the stocking of juvenile vessels with 25 individuals each.

Juveniles were fed on *A. franciscana* at a density of three freshly hatched nauplii per mL, 2 *A. franciscana* /mL and 1 *A. franciscana* /mL for the first 3 days, respectively, along with artificial food (below). Administration of *A. franciscana* was ended after the 3rd day. To obtain females derived from the diatom-induced sex change described above, two groups of juveniles were allotted, based on the artificial food type given. The food types given were: (1) composed dry food (CTRL−) and (2) composed dry food added with the benthic diatom *Cocconeis scutellum parva* (CTRL+). The food CTRL− was composed of three dry ingredients mixed in equal quantities (Zupo et al. 2007), i.e., dry *A. franciscana*, dry *Spirulina* sp. and “Microperle” (micro-encapsulated supplementary feed for marine invertebrates); they were all provided by SHG (Ovada, Italy). In addition to the ingredients of CTRL−, CTRL+ contained 50% in weight of the diatom *Cocconeis scutellum parva*, known for causing regression of androgenic glands in young juveniles (Zupo and Messina 2007; Zupo et al. 2014). This diatom was used to trigger the production of young females.

The food CTRL− was administered to produce a higher abundance of males, according to the physiology of this species (Zupo 2000) while CTRL+ treated postlarvae supposed to produce a larger abundance of females. Food was offered at the rate of 3.50 mg/day per 400 mL of seawater (38 psu) for the first 15 days. The quantity of food given was increased up to 7.0 mg/day in the next days, to guarantee it was provided ad libitum. The survivors were counted daily and then transferred into new culture solution in clean vessels, using a Pasteur pipette. Exuviae were collected regularly and observed using a microscope (Leica DMLB) for any sign of maturation, i.e., presence/absence of *A.M*. and *A.I*.. Juveniles were fixed in 70% alcohol when they reached the size of 7–8 mm and were assumed to be sexually mature. Two individuals were sampled from each (CTRL− and CTRL+) treated juvenile culture vessel at 5-days interval (0, 5, 10, 15, 20, 25 days, respectively) and fixed in 70% alcohol to investigate ontogenetic changes of gonopores.

### Analysis of sex

In total, 50 individuals (10 mature, 40 immature) were analysed for the presence/absence of *A.M*. and their gonopores were observed as well. Twelve additional individuals were taken for analysis of ontogenetic changes of gonopores. Their total body length was measured under a complanar apochromatic macroscope (Leica Z16-APO) using a millimetric paper. Individuals fixed in 70% alcohol were stained in 2% methylene blue solution and immediately observed for the location and shape of gonopores. Males were characterised by the presence of gonopores at the far proximal end of the coxa of the fifth pair of pereiopods, whilst the gonopores of females were located at the far proximal end of the third pair of pereiopods (Bauer 2004). In addition, mature individuals were also observed and their second pleopods were collected to detect the absence/presence of *A.M*. using bright-field light microscopy (Leica DMLB). Presence of an *A.M*. on the pleopod II corresponds to males, while its absence characterises females.

The lengths of exopodite, endopodite, basipodite and *A.I*. of each of the 40 immature individuals as well as their body size were measured. These individuals belong to the same age group and they have similar sizes. All measurements were made on images of the shrimps obtained by a Leica Z16-APO macroscope equipped with a computerised system of image analysis.

### Data analysis

The ratio number of females/total number of mature individuals (*F*/*Mat*) was calculated for each group. The ration number (*F*/*Mat*) between CRTL+ and CTRL− were compared by means of the *z* test on proportions. The ratio females/total number of mature individuals was chosen to make the experiment independent from the number of immature. Large values for *F*/*mat* ratios indicate apoptogenic activity.

Regression curves were obtained for each couple of biometric parameters and, in particular, the lengths of *A.I*., basipodites, endopodites and exopodites were compared to the total lengths of males and females. Slopes and correlation coefficients were computed to test the relationships between the size of shrimps and the lengths of basipodite, exopodite, endopodite and *A.I*.. Morphometric data were separately computed for males and females, to allow a comparison of the two sexes. We compared the regression slopes of males and females to test the null hypothesis (*H*_o_):

$${\text{Bf}}={\text{Bm,}}$$

where Bf is the slope for females, and Bm is the slope for males.

To perform this analysis, we used *t* test and analysis of covariance (ANCOVA). It compares the independent variable *X* (here, total length) and the dependent variable *Y* (basipodite, exopodite, endopodite and *A.I*.) between two groups. The purpose of ANCOVA, in our case, is to compare two or more linear regression lines. It is a way of comparing the *Y* variable among groups, while statistically controlling for variation in *Y* influenced by the values assumed by the *X* variable. Two null hypotheses are tested in ANCOVA. The first is that the slopes of the regression lines are all the same. If this hypothesis is not rejected, the second null hypothesis is tested, that the *Y*-intercepts of the regression lines are all the same. We performed a power analysis on the sample size needed for ANCOVA using the method proposed by Borm et al. (2007).

## Results

### Feeding experiments

Comparison of the effects between the two juvenile groups on sex change showed that CTRL+ produced a higher *F*/*Mat* ratio (67%) as compared to CTRL− (26.51%). Treatment CTRL+ showed apoptogenic activity, yielding a number of females that is significantly different from CTRL− (*z* test, *p* = 0.03).

### Morphological observations

**Fig. 1:**
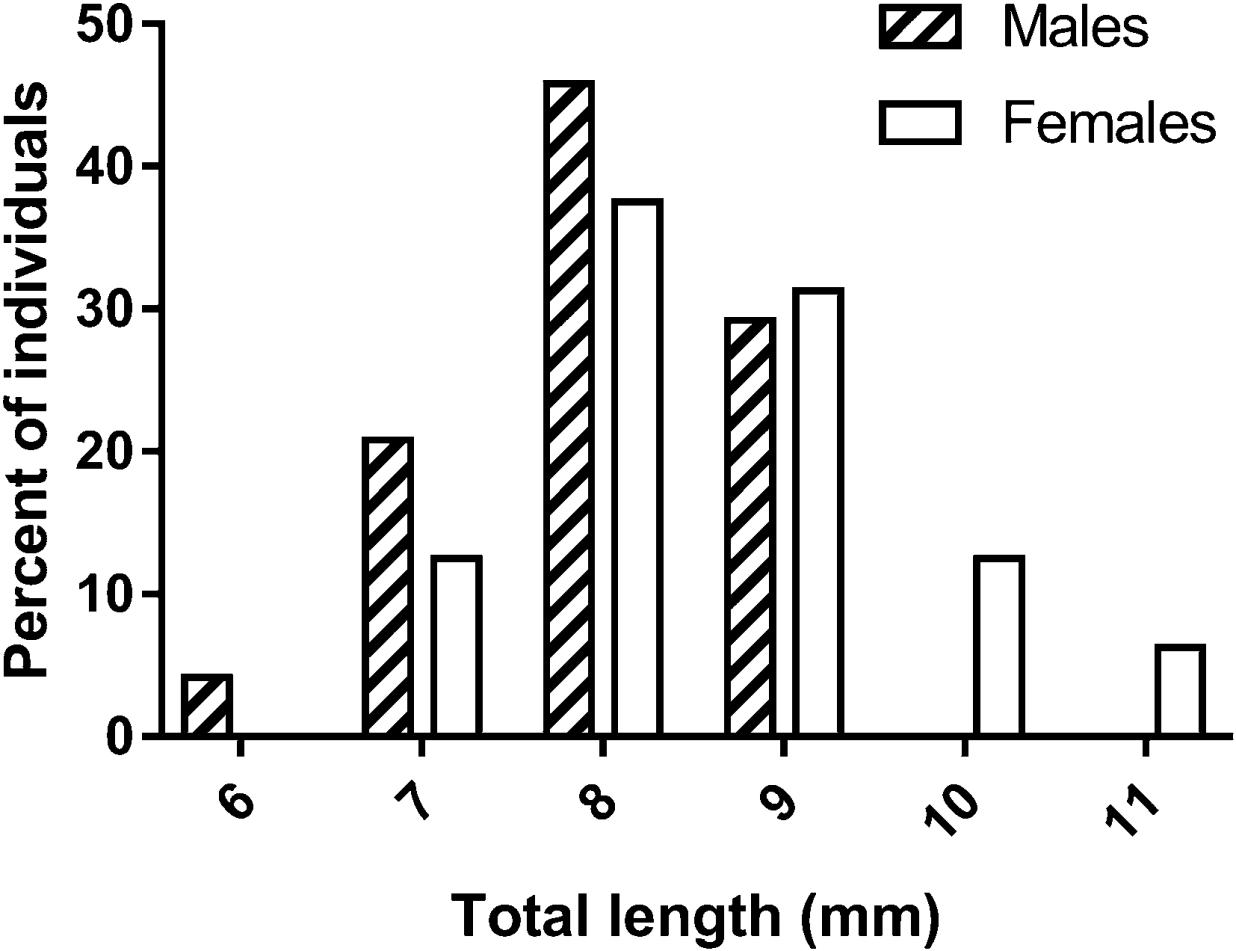
Size frequency distribution of males and females of *H. inermis* according to their sex, determined by means of the shape and position of gonopores

**Fig. 2:**
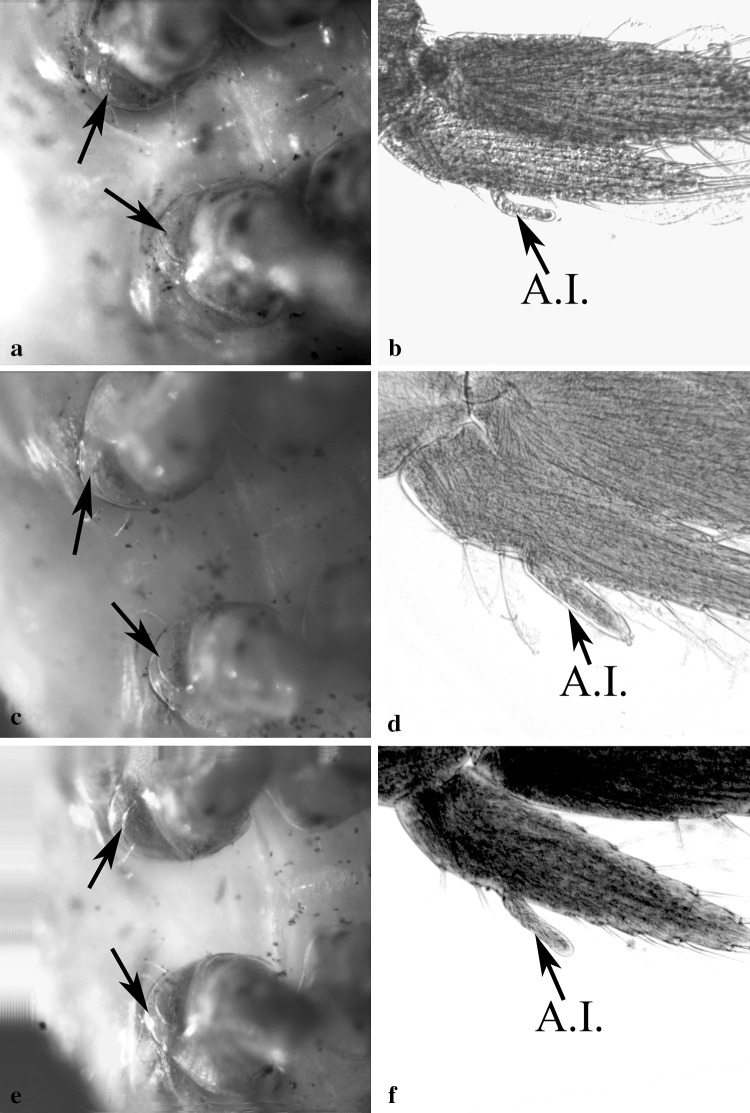
Male gonopores of *H. inermis*, observed under a macroscope (left) and shape of the second pleopod of the same individuals observed under bright-field light microscopy (right). Arrows indicate the location of the gonopores on the coxa of the fifth pair of pereiopods (**a, c, e**). The corresponding sexual appendices present on the endopodite of the second pleopod are also indicated (**b, d, f**). *Appendix masculina* was absent in all the immature males. A.I.: *appendix interna* (not to scale)

**Fig. 3:**
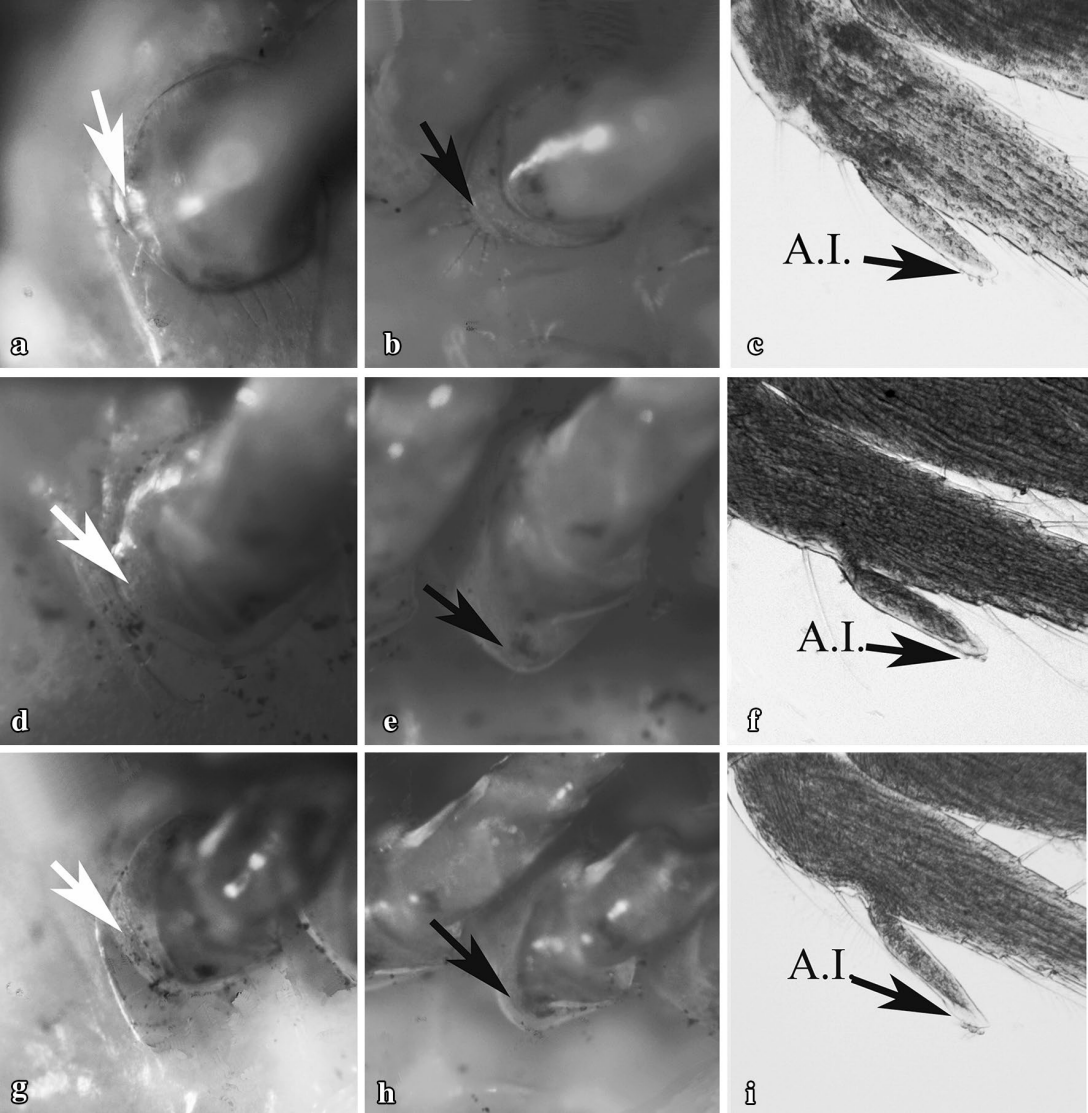
Female gonopores of *H. inermis*, observed under a macroscope (left two figures) and shape of the second pleopod of the same individuals observed under bright-field light microscopy (right figures). Black arrows indicate the location of gonopores on the coxa of the third pair of pereiopods (**b, e, h**); the corresponding sexual appendices present on the endopodites (second pleopod) of same individuals are also indicated (**c, f, i**). Male gonopores located on fifth pereiopods were absent in the individuals identified as females (white arrows **a, d, g**). A.I.: *appendix interna* (not to scale)

**Fig. 4:**
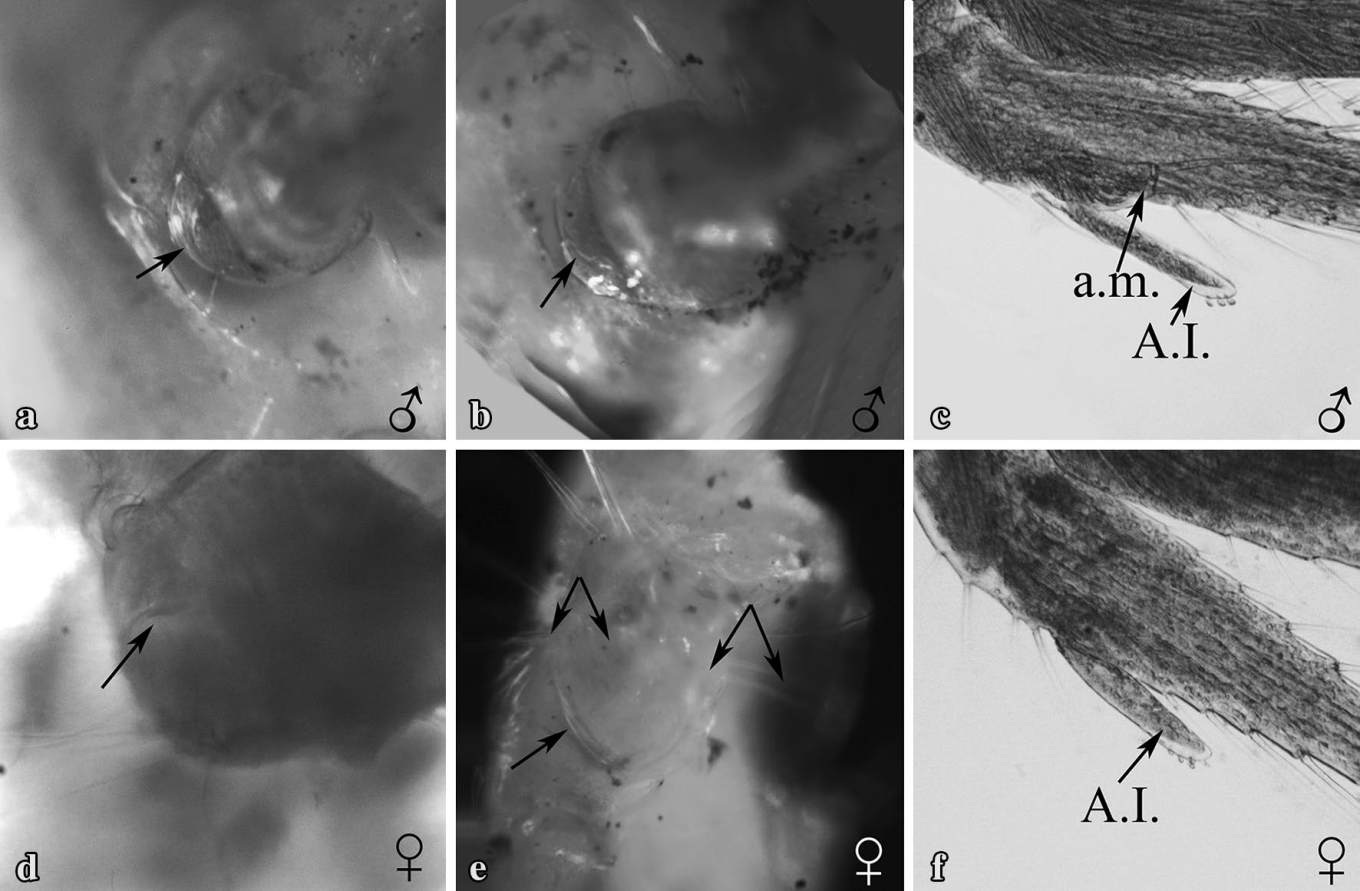
Location of male (**a, b**) and female (**d, e**) gonopores observed in mature individuals. The A.M. can be seen very prominently in mature males (**c**) while it is absent in females (**f**); double arrows (**e**) indicate the egg guiding setae as seen in mature females. The sex of each individual is indicated at the lower right corners. A.M.: *appendix masculina*; A.I.: *appendix interna* (not to scale)

Gonopores of all examined individuals were located either at the proximo-medial end of the third or the fifth pair of pereiopods, according to the sex. None of the examined individuals showed the simultaneous presence of male and female gonopores. We recorded 16 females and 24 males out of 40 immature individuals, classified by detecting the presence, position and shape of gonopores. Males and females were sorted according to the size. The size frequency distribution of the two sexes, according to the total length of shrimps, followed a bell-shaped trend (Fig. 1). Most small individuals (between 6 and 7 mm) were males and larger specimens were all females (10 and 11 mm of total length). Intermediate sizes (between 8 and 9 mm) were represented by both sexes. Males were characterised by the presence of gonopores at the proximal end of the coxopodite of the fifth pair of pereiopods, whilst in females the gonopores were located at the proximal end of the coxopodite of the third pair of pereiopods. Male gonopores were easily identified and they appeared like a bulge, with their opening located on the medial side of the fifth pereiopod coxae (Fig. 2a, c, e). Female gonopores appeared U-shaped, present on the proximo-medial side of the third pereiopod coxae (Fig. 3b, e, h). A cup-like structure was also present, distal to the female gonopore (Fig. 4d, e; as indicated by the arrows). Furthermore, examination for the presence of male gonopores on the fifth pair of pereiopods (Fig. 3a, d, g) on those individuals designated as females by the gonopore technique, revealed the absence of these structures.

**Fig. 5:**
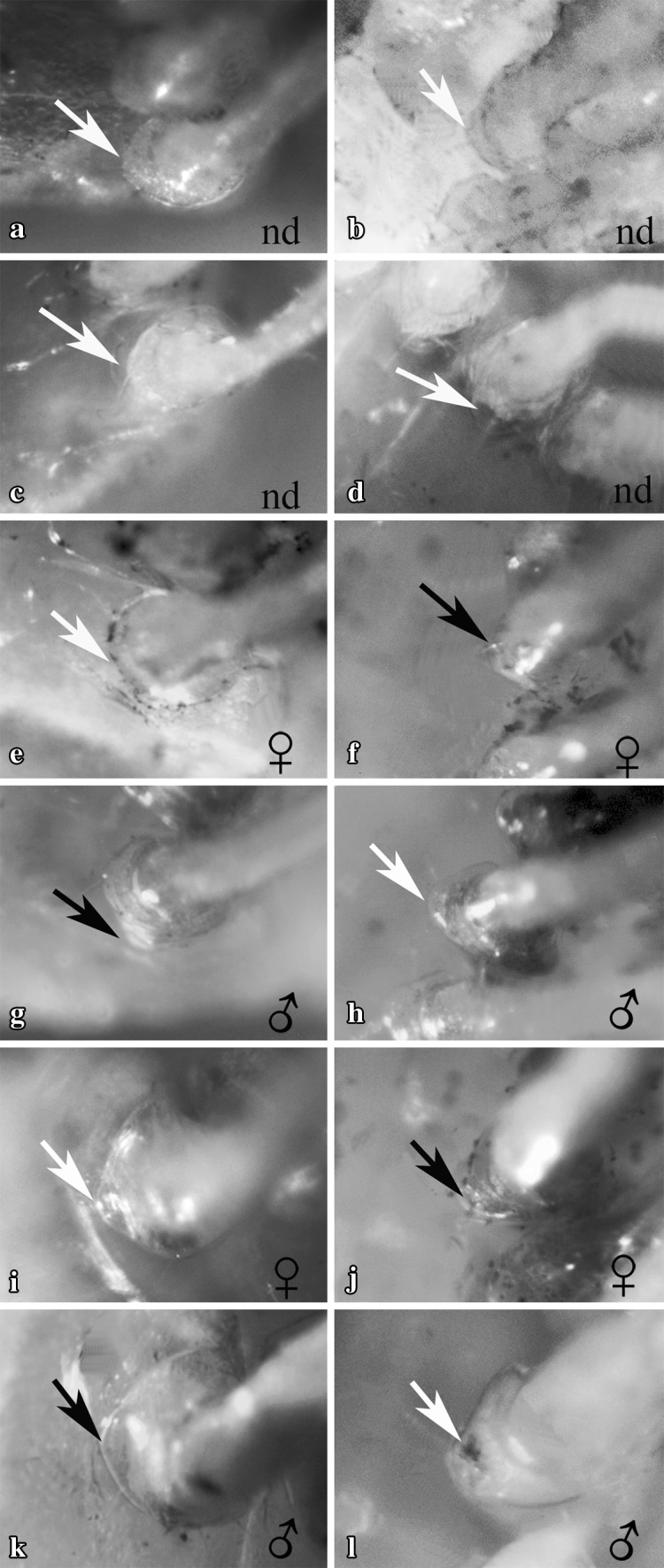
Ontogenetic changes of gonopores in juveniles of *H. inermis*. Arrows indicate the possible location sites of gonopores in 0-day- (**a, b**) and 5-day (**c, d**)-old juveniles. From the 10th day onwards, the gonopores were visible under a macroscope. Fifth pereiopods (**e, g, i, k**) and third pereiopods (**f, h, j, l**) observed under a macroscope; white arrows indicate the absence of gonopores (**e, h, i, l**); black arrows indicate the presence of gonopores (**f, g, j, k**). Individuals of various ages are shown: 10 days old (**e, f**); 15 days old (**g, h**); 20 days old (**i, j**) and 25 days old (**k, l**). The sex of each individual is indicated at the lower right corners. *n.d*.: not defined (not to scale)

The gonopore ontogenetic changes in juveniles showed that, from day 10 onwards, the gonopore structure and location is visible using a macroscope (Fig. 5f, g, j, k). However, it was impossible to detect gonopore locations up to the 5th day of juvenile development, using a macroscope (Fig. 5a–d). Individuals from 0 to 5 days old were too small to handle and not easily observable using a macroscope.

### Biometric data

**Fig. 6:**
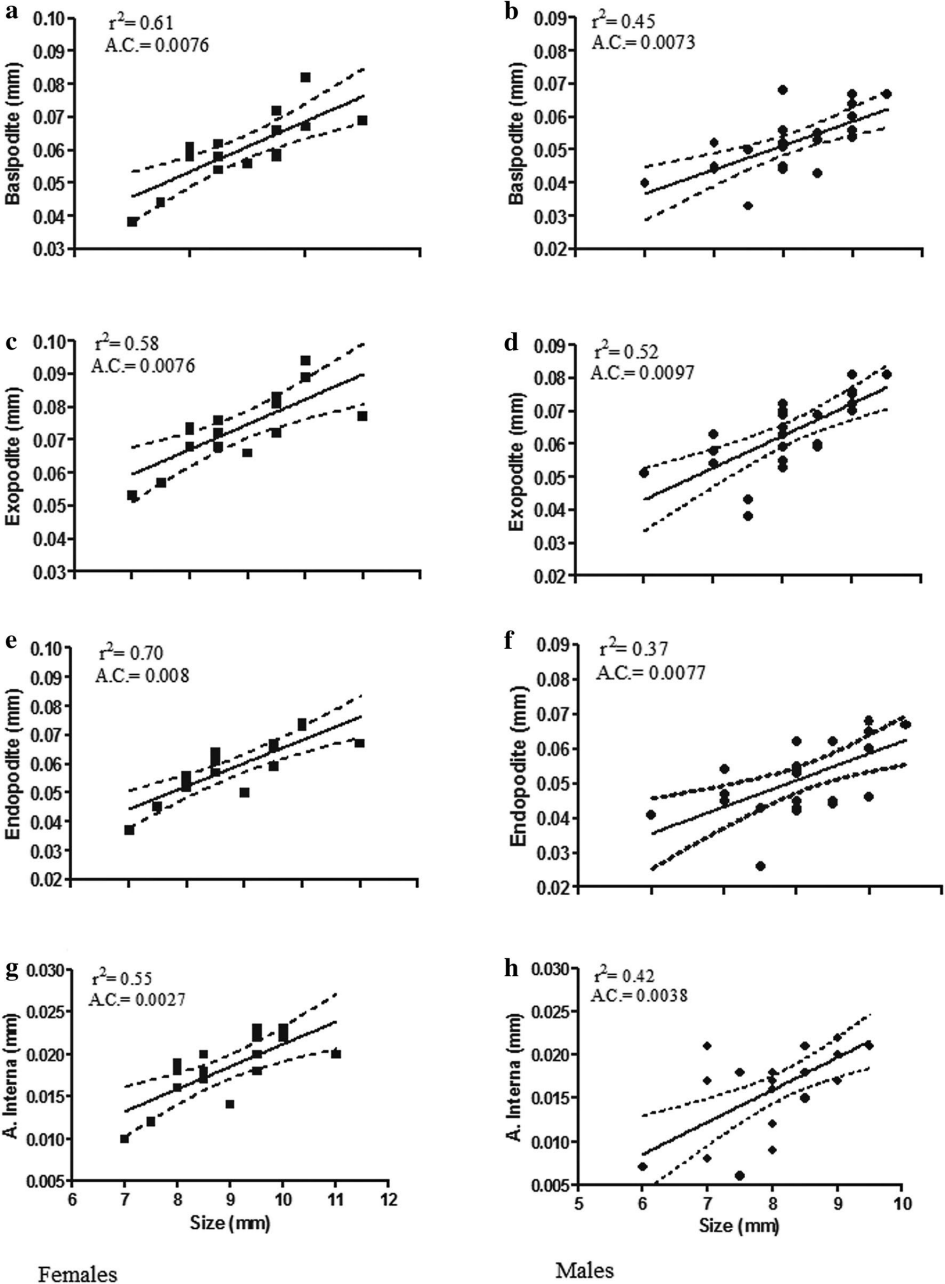
Relationships between total body length (excluding antennae) of *H. inermis* and the length of elements of pleopod II: basipodite (**a, b**); exopodite (**c, d**); endopodite (**e, f**); *appendix interna* (**g, h**), in females (left plots) and males (right). A.C.: angular coefficient (slopes)

The sample size required to achieve a power 1 − *β* = 0.8, effect size = 0.008, at level *α* = 0.05, for each group was 14. A significant difference in the total length was observed among males and females; *t* (38) = 2.498, *p* = 0.02. Males were found to be slightly (9.23%) smaller than females. The main biometric features did not differ significantly between males and females, according to the results of ANCOVA (*p* > 0.05). However, a higher linear correlation was found among the female biometric measures, when regressed against size, than among the male biometric features. The basipodites of females ranged from 0.038 to 0.082 mm in females and their sizes were slightly lower in males, but in the latter case the correlation (*R*^2^) with total length was 0.45 (Fig. 6b), whilst in females the same correlation reached 0.61(Fig. 6a). Similarly, the size of exopodites in females ranged from 0.053 to 0.094 mm, whilst in males it ranged from 0.051 to 0.081 mm. However, the correlation (*R*^2^) of these measures with the total length accounts for 0.58 in females and 0.52 in males (Fig. 6c, d). The endopodites ranged from 0.037 to 0.074 mm in females and from 0.026 to 0.68 mm in males, but the correlation (*R*^2^) with size accounts for 0.70 in females and 0.37 in males (Fig. 6e, f). Finally, the size of the *A.I*. (Fig. 6g, h) ranged from 0.010 to 0.023 mm in females (*R*^2^ = 0.55) and from 0.07 to 0.023 in males (*R*^2^ = 0.42).

## Discussion

### Gonopore morphology

Our results showed that the sex of *H. inermis* can be identified according to the presence of either male or female gonopores. The location of gonopores on the third pair of pereiopods in females and on the fifth pair of pereiopods in males is in line with the previous literature on caridean shrimps (Mascetti et al. 1997; Espinoza-Fuenzalida et al. 2008; Terossi et al. 2008; Tóth and Bauer 2007, 2008; Manjon-Cabeza et al. 2011). The shape and structure of male gonopores in our study are similar to those observed by Espinoza-Fuenzalida et al. (2008) in *Hippolyte williamsi*. Female gonopores in *H. inermis* are characterised by the presence of a flap, just distal to the gonopore opening, at the proximal end of the third pair of pereiopod coxae. This finding is in accordance with observations made by previous authors in snapping shrimp *Synalpheus* sp. (Tóth and Bauer 2007, 2008). From the gonopore ontogenetic analysis, it is evident that 10-day old juveniles can be sexed using the gonopore technique (Fig. 5), whilst, in case of standard method of sex assessment, one must wait until the sexual maturity of juveniles that usually takes 28–30 days. As such, the gonopore technique is a reliable and cheap method to study sex reversal with lesser time.

### Sexual systems in Caridea

There is a substantial range of variation in the sexual systems adopted by carideans viz. gonochory, protandry, protandric simultaneous hermaphroditism and various forms of sequential hermaphroditism (Bauer 2000; Zupo and Maibam 2010). *H. inermis* probably represents the most debated case of protandry. Reverberi (1950) described this species as protandric, which was later confirmed by Zupo (1994, 2000, 2001). In contrast, Cobos et al. (2005) argued this species to be gonochoristic, after demonstrating no evidence of gonadal transition through an ovotestis (Berreur-Bonnefant and Charniaux-Cotton 1965). Finally, Zupo et al. (2008) experimentally demonstrated the change of sex of various individuals in the laboratory, confirming previous conclusions by Reverberi (1950). In this peculiar species, the sex change occurs very quickly, during a single moult cycle, after the complete disruption of the testes. Therefore, the presence of intersexes, indicated by Bauer (1986) as a reliable sign of sex change, was never found in this species. This study, in contrast, indicates that the contemporaneous presence of male and female gonopores is not an indispensable feature of sex-reverting decapods (Sagi et al. 1997), since a fast sex change, as observed here, may be compatible with a hermaphroditic life strategy.

### Gonopores in *H. inermis*

As above stated, an ovotestis never appears during the sex reversal process in *H. inermis*, since the ovary is produced starting from a few germ cells (Reverberi 1950). Concurrent presence of male and female gonopores was never observed in the present study, unlike findings for such species as *Synalpheus paraneptunus* where intersex individuals have been observed (Tóth and Bauer 2008). Each individual was characterised by either the presence of a pair of male or female gonopores indicating the absence of intersexes. A gonochoristic sexual system has been demonstrated in congeneric species viz. *H. williamsi* (Espinoza-Fuenzalida et al. 2008), *H. obliquimanus* (Terossi et al. 2008), and *H. niezabitowskii* (Manjon-Cabeza et al. 2011). As expected, the number of females in the treatment CTRL+ was higher than in the CTRL−, indicative of the process of sex reversal triggered by the apoptogenic compounds present in the CTRL+ food (Zupo and Messina 2007). Therefore, this study confirms that the addition of *Cocconeis scutellum* to the diet of *H. inermis* triggers the production of early females, as happens in natural populations during the spring recruitment period (Zupo 1994). In contrast, the absence of these diatoms, as revealed by the CTRL− treatment and observed in the field during the fall reproduction, produces mainly males, undergoing a sex-reversal process only after 1 year (Zupo 2000).

A higher variability of male biometric measures, as compared to female measures, remains to be explained. In fact, we observed that the lengths of basipodites, exopodites and *A.I*. of males and females are correlated to body size, and their size does not vary significantly between the two sexes, but the correlation coefficient is constantly higher in females in respect to males. In particular, the most variable feature is the length of the *A.I*.. This is closely related to the size of females, whilst it is largely variable in males, when they still miss an *A.M*. (it will be developed in future, since the examined individuals, identified by means of the gonopore technique, were still immature).

Taking into account that females (all *beta* females, due to the experimental constraints above mentioned) were generated by the early disruption of the juvenile testes, while the males were naturally produced by feeding on a food not containing apoptogenic diatoms, we can assume that young males are exposed to environmental influences, whilst female physiology is dependent on the maturation of the ovaries. These observations are in agreement with Zupo and Messina (2007), observing that the administration of apoptogenic compounds produces, besides sex reversal, an initial period of slower growth followed by rapid maturation of ovaries. We cannot exclude, however, that some males we analysed might be helpers in a non-reproductive phase (Tóth and Bauer 2007), still competent to develop into females or reproductive males and, in this case, the different physiological destiny could produce the larger variability in the biometric relationships. It is evident that the life strategy of *H. inermis* (Zupo 1994) should be taken into account to explain the evolution and maintenance of its protandric hermaphroditism strategy (Bauer 1996).

Protandric hermaphrodites are often considered to evolve due to the “size advantage hypothesis” Warner (1975), but larger individuals are also more easily predated on the leaves of *P. oceanica* (Zupo et al. 2008). Therefore, *H. inermis* developed the ability to produce beta (small) females in spring, taking advantage of the large abundance of trophic resources to obtain a reproductive burst, since several small females are promptly introduced in natural stocks. The “size advantage”, in contrast, is exploited in fall, when an all-male generation is produced, able to generate large (*alpha*) females in the next reproductive season (Zupo 1994). In this way, the species takes advantage of both strategies, thanks to the double periods of reproduction and the two different sexual systems, and this guaranteed the stability of natural populations in the leaf stratum.

In conclusion, the technique here developed allows for early identification of the sex in small shrimps and the results obtained are in agreement with those based on the presence/absence of an *A.M*.. In addition, this study confirms previous observations indicating that *H. inermis* undergoes a peculiar process of sex reversal, very rapid and proceeded by the apoptotic disruption of the testes and the androgenic gland (Zupo and Messina 2007). For this reason, not only primary sexual characters that are considered typical of hermaphroditic species during transitional stages (e.g., an ovotestis; Cobos et al. 2005; Tóth and Bauer 2007) are never produced, but also the secondary sexual characters commonly observed in transitional stages of hermaphroditic malacostracans (biometric changes in the size of gonopores as well as reduced spination of the *A.M*. and cincinnuli on pleopod I, with development of the female incubatory flanges) are not found. On the whole, all these findings are in agreement, indicating an atypical process of sexual inversion (Reverberi 1950), which may shed light on general patterns of sex assessment and change in decapod crustaceans.
